# Rivaroxaban in heart failure patients with left ventricular thrombus: A retrospective study

**DOI:** 10.3389/fphar.2022.1008031

**Published:** 2022-10-07

**Authors:** Qian Zhang, Zhongfan Zhang, Haikuo Zheng, Ming Qu, Shouping Li, Ping Yang, Daoyuan Si, Wenqi Zhang

**Affiliations:** ^1^ Department of Cardiology, China-Japan Union Hospital of Jilin University, Changchun, China; ^2^ Department of Gastroenterology, Endoscopy Center, China-Japan Union Hospital of Jilin University, Changchun, Jilin, China

**Keywords:** heart failure, left ventricular thrombus, anticoagulation management, rivaroxaban, vitamin K antagonists

## Abstract

**Background:** The role of rivaroxaban in patients with heart failure (HF) combined with left ventricular (LV) thrombus remains unknown in current guideline-directed anticoagulant therapy. The aim of this study was to investigate the impact on clinical outcomes of rivaroxaban compared to vitamin K antagonists (VKAs) in patients with HF combined with LV thrombus.

**Methods:** We retrospectively extracted clinical, echocardiographic and follow-up data of HF patients (all classifications) admitted at China-Japan Union Hospital of Jilin University from January 2017 to June 2021. A total of 198 patients with HF were identified with LV thrombus by echocardiography, 78 of them were managed with VKAs, 109 with rivaroxaban.

**Results:** The median follow-up was 17.0 months (interquartile range: 6.0–24.0 months). High rates of major cardiovascular adverse events (MACEs) were observed in both the rivaroxaban and VKAs groups (49.5% vs. 57.7%). However, rivaroxaban *versus* VKAs observed a decrease in MACEs (adjusted HR:0.636; 95%CI:0.418–0.970; *p* = 0.035) and systemic embolism (4.6% vs. 12.8%; adjusted HR:0.318; 95%CI:0.108–0.933; *p* = 0.037; Gray’s test *p* = 0.041) but was not found to have a benefit with regard to LV thrombus resolution (59.6% vs. 70.6%; adjusted HR: 1.303; 95% CI:0.898–1.890; *p* = 0.163; Gray’s test *p* = 0.073). Additionally, there was no significant between-group difference in the rate of International Society on Thrombosis and Hemostasis (ISTH) bleeding events.

**Conclusion:** Our data found that in populations with HF combined with LV thrombus, the overall prognosis in both the rivaroxaban and VKAs groups was catastrophic. Although rivaroxaban improved the prognosis to some extent, a considerable need remains for new treatments to improve their clinical course.

## Introduction

Despite the considerable progresses made during the last few years in heart failure (HF) management, there remain many aspects of HF therapy in clinical practice which lack strong trial evidence (Siliste et al., 2018; Maggioni and Andreotti, 2019). Anticoagulation therapy in patients with HF combined with left ventricular (LV) thrombus constitutes one of these examples (Ezekowitz et al., 2017). Patients with HF are at increased risk for the development of left ventricular (LV) thrombus owing to stasis of blood in hypokinetic or akinetic regions (Zannad et al., 2013; Lin et al., 2021; Levine et al., 2022). Meanwhile, HF patients with LV thrombus tend to experience higher rates of adverse events than those without LV thrombus. Currently, treatment with oral anticoagulants (Either warfarin or a direct oral anticoagulant) has emerged as a possible strategy for improving outcomes in this area (Ezekowitz et al., 2017). However, the guidelines also acknowledge that this recommendation was made on the basis of the lack of trial evidence and mechanism of action (Ezekowitz et al., 2017). Since previous large studies have not specifically focused on HF patients with LV thrombus (Homma et al., 2012; Zannad et al., 2018), the prognosis in this population remains mostly unknown under the current guideline-directed medical therapy. A recent retrospective study reported that under the anticoagulation management of vitamin K antagonists (VKAs), the prognosis of HF complicated with LV thrombus was catastrophic, with a high rate of 20% 1-year systemic embolism and mortality (Lemaître et al., 2021). However, the prognosis of the anticoagulation management of direct oral anticoagulants (DOACs) has not been reported in this area.

Rivaroxaban, as a type of DOACs, has been observed with more positive results than VKAs in other clinical settings (Sherwood et al., 2014; van Es et al., 2014). Given the lack of data on the application of rivaroxaban in HF patients with LV thrombus, we designed a retrospective study to investigate the impact on clinical outcomes of rivaroxaban compared with VKAs under the current guideline-directed anticoagulant management in HF patients with LV thrombus.

## Materials and methods

### Study population

This study is a retrospective observational study from a large tertiary referral center. We conducted a computerized search for all HF patients (all classifications) admitted at China-Japan Union Hospital of Jilin University from January 2017 to June 2021. HF patients with LV thrombus on echocardiography, regardless of underlying disease causing the HF, were screened, and only patients with confirmed LV thrombus by two independent cardiologists and with documented routine follow-up were included in this study. In the event of disagreement between cardiologists reviewing the LV thrombus images, the images were submitted to another independent cardiologist for confirmation of the final review. We extracted information from the available medical record, and retrieved information regarding all-cause mortality, rehospitalization for cardiovascular events, systemic embolism, or bleeding events from routine clinical follow-up after LV thrombus diagnosis. Patients who had not yet documented a review event in the medical record were contacted individually by telephone to finalize the accuracy. Detailed definitions of HF and LV thrombus echocardiographic evaluation were presented in the Supplementary Appendix. Written consent was waived due to the minimal patient risk and the retrospective study design. The study protocol was reviewed and approved by the ethical review board of China-Japan Union Hospital of Jilin University.

### Antithrombotic management

According to current guidelines for the management of heart failure (Ezekowitz et al., 2017), anticoagulation management with either a VKAs (warfarin) or DOACs (mostly rivaroxaban and a few dabigatran) was initiated when LV thrombus was diagnosed in HF patients in our center. Periodic re-evaluation of LV thrombus status was performed to adjust anticoagulation duration. In patients at high risk of bleeding (i.e., previous history of bleeding, renal failure, or anemia), the dose of anticoagulant was individualized according to the guidance from responsible physician. Whether or not to administer antiplatelet therapy was determined by the responsible physician at the time, depending on the patient’s underlying disease and the management strategy for that disease.

### Clinical covariates

All clinical covariates including demographic data, past medical history, underlying disease, laboratory biochemical parameters, and echocardiographic data were collected through medical records or by interaction with patients or their family members. Those clinical covariates were associated with the prognosis of thrombosis and heart failure in previous studies (Zannad et al., 2018; Lemaître et al., 2021). To address and minimize bias in retrospective study design, data collection was standardized with precise definitions for each clinical covariate and measure. Since the final missing data were small (<5%), the missing data in clinical covariates were imputed using the mean or mode.

### Endpoints and definitions

In this study, we mainly investigated the three endpoints: LV thrombus resolution, major adverse cardiovascular events (MACEs) and bleeding. LV thrombus status was defined based on the method of Lattuca et al. (2020). LV thrombus resolution was defined as the complete disappearance of LV thrombus on all echocardiographic views at the last available follow-up visit. LV thrombus persistence was defined as the visibility of thrombus on all echocardiographic views at the last available follow-up visit. Major adverse cardiovascular events (MACEs) were defined as a composite of all-cause mortality, systemic embolism, and rehospitalization for cardiovascular events following an LV thrombus diagnosis. Bleeding events were defined as major bleeding, clinically relevant non-major bleeding and minor bleeding according to International Society on Thrombosis and Hemostasis (ISTH) criteria (Schulman and Kearon, 2005; Kaatz et al., 2015). All endpoint events were reviewed by a clinical academic group independent of this study based on pre-specified event definition criteria. The detailed adjudication procedures and definitions of endpoint events were shown in the Supplementary Appendix.

### Statistical analysis

Baseline characteristics were described as continuous or categorical variables. Continuous variables were represented as mean (SD) or median (IQR), and comparisons between groups were performed using Student’s t-test or Mann-Whitney *U* test, as appropriate. Categorical variables were presented as number (%), and comparisons were performed using the χ2 or Fisher’s exact test as appropriate.

The influence of anticoagulant types (Rivaroxaban vs. VKAs) on outcomes in patients with HF combined with LV thrombus was assessed by hazard ratios and their 95% confidence intervals (CIs) using Cox proportional hazard regression models. All clinical covariates were assessed by univariate Cox proportional hazards regression to determine whether they were significantly associated with outcomes. Then, covariates with *p* ≤ 0.05 in the univariate models were included in the multivariate Cox proportional hazards regression models to identify the independent effect of anticoagulant types on outcomes (Agoritsas et al., 2017). Meanwhile, considering the analytical effect of the number of events on the multivariate proportional hazards regression models, we used the principle of EPV = 10 for determining the number of covariates to improve the accuracy and precision of the multivariate models (the covariates incorporated in each model were detailed in the in the Supplementary Table SA6) (Peduzzi et al., 1995). Validity of the proportionality assumption was verified by a visually examining Schoenfeld-type residual plot or a weighted residuals test. Kaplan-Meier curves and log-rank test were used to examine differences in the rate of LV thrombus resolution across study groups.

Additionally, considering the impact of higher mortality in the heart failure population on other non-mortality endpoints, we used the Fine-Gray models as the sensitivity analyses to assess the robustness of these non-mortality study findings (Scrucca et al., 2010). These sensitivity analyses were conducted after adjusting for the competing risk for mortality. Finally, exploratory analyses were performed in various subgroups to investigate the influence of anticoagulant types on outcomes in each subgroup. The exploratory endpoints included LV thrombus resolution, MACEs, all-cause mortality, systemic embolism, rehospitalization for cardiovascular events. We used the Cox proportional Hazard joint test to assess the interaction between treatment effect and these subgroup characteristics. All analyses were performed using R version 4.1.1 (R Foundation for Statistical Computing, Vienna, Austria) and SPSS version 24.0 (IBM Corp, Armonk, NY, United States) with a 2-sided *p* value < 0.05 indicating statistical significance.

## Results

### Patients

**FIGURE 1:**
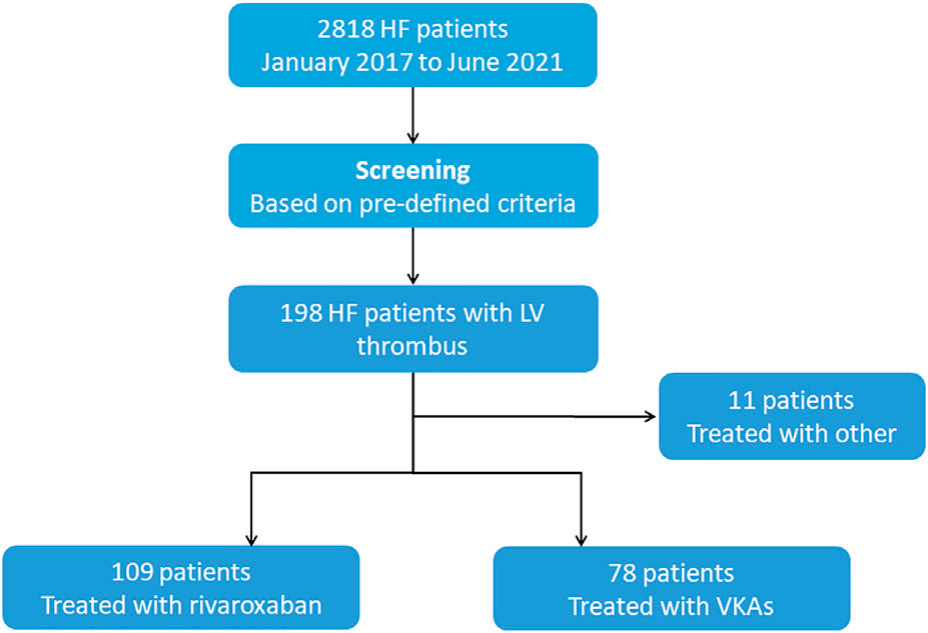
Study Chart Flow. HF = heart failure; VKAs = vitamin K antagonists.

During the study period, a total of 198 patients with HF were identified with LV thrombus by echocardiography, 78 of them were managed with VKAs, 109 with rivaroxaban (Figure 1). Median anticoagulation durations were 4.0 months (IQR:1.0–10.0 months) in the VKAs group and 2.0 months (IQR: 1.0–7.0 months) in the rivaroxaban group, respectively. The baseline characteristics of patients with confirmed LV thrombus were shown in Table 1. At baseline, compared to the VKAs group, patients with rivaroxaban had a higher proportion of ischemic heart disease accompanied by higher levels of haemostatic markers (Fibrinogen), larger LVEDD, and lower LVEF. Other baseline characteristics including age, sex, antiplatelet therapy and coronary risk factors did not differ significantly between the two groups. A more exhaustive review of the study population was included in the supplemental appendix, including the types of underlying disease (Supplementary Table SA1), and antiplatelet drug administration (Supplementary Table SA2).

**TABLE 1:** Clinical characteristics in heart failure patients with LVT.

Baseline characteristics	VKAs (*n* = 78)	Rivaroxaban (*n* = 109)	Other (*n* = 11)	p value^a^
Male, n (%)	66 (84.6)	85 (78.0)	11 (100.0)	0.257
Age, yrs	63.0 (54.5–71.0)	64.5 (54.2–70.8)	64.0 (49.0–67.0)	0.882
Body mass index, kg/m^2^	24.0 (21.0–26.3)	24.3 (22.1–27.0)	24.6 ± 2.4	0.292
Prior SSE, n (%)	28 (35.9)	33 (30.6)	1 (9.1)	0.444
Hypertension, n (%)	33 (42.3)	45 (41.3)	3 (27.3)	0.889
Diabetes mellitus, n (%)	21 (26.9)	57 (30.5)	1 (9.1)	0.371
Current smoker, n (%)	41 (52.6)	42 (38.5)	5 (45.5)	0.057
Chronic heart failure, n (%)	39 (50.0)	59 (54.1)	7 (63.7)	0.577
Ischemic heart disease, n (%)	61 (78.2)	97 (89.0)	6 (54.5)	0.045
Atrial fibrillation, n (%)	6 (7.7)	7 (6.4)	0 (0)	0.736
Antiplatelet therapy	63 (80.8)	80 (73.4)	7 (63.6)	0.241
Creatinine clearance, mL/min/1.73 m^2^	70.6 (53.2–87.9)	63.6 (55.3–87.9)	80.1 ± 26.4	0.638
WBC, ×10^9^	8.2 (6.8–10.5)	8.3 (6.6–10.9)	9.0 (6.0–11.7)	0.981
Hemoglobin, g/L	144.0 (128.0–158.0)	146.0 (130.3–157.0)	142.0 ± 22.7	0.809
NT-proBNP, pg/mL	5530.0 (1465.0–7910.0)	4580.0 (1752.5–9305.0)	6060.0 (1490.0–10000.0)	0.904
D-dimer, mg/L	1.4 (0.7–3.0)	1.6 (0.7–3.0)	1.7 (1.0–3.0)	0.642
Fibrinogen, mg/dl	4.0 (3.2–4.2)	3.5 (2.8–4.0)	4.3 ± 1.7	0.028
LV ejection fraction, %	39.0 (31.0–51.0)	35.0 (27.0–44.0)	35.4 ± 13.5	0.005
LVEDD, mm	51.7 (45.5–59.7)	55.9 (49.1–63.5)	55.2 ± 11.2	0.042
LV aneurysm,n (%)	16 (20.5)	18 (16.5)	1 (9.1)	0.484
Mitral regurgitation area, cm^2^	2.5 (0–5.6)	3.6 (1.8–6.3)	2.9 (0–4.2)	0.113
Thrombus size, mm^2^	256.0 (136.5–474.5)	284.0 (177.3–498.5)	387.1 (130.5–666.0)	0.589

a P value was for rivaroxaban group as compared with VKAs group.

Abbreviations: WBC, white blood cell count; MPV, mean platelet volume; LV ejection fraction:left ventricular ejection fraction; LVEDD, left ventricular end-diastolic dimension; LV aneurysm:left ventricular aneurysm.

### Left ventricular thrombus resolution

**FIGURE 2:**
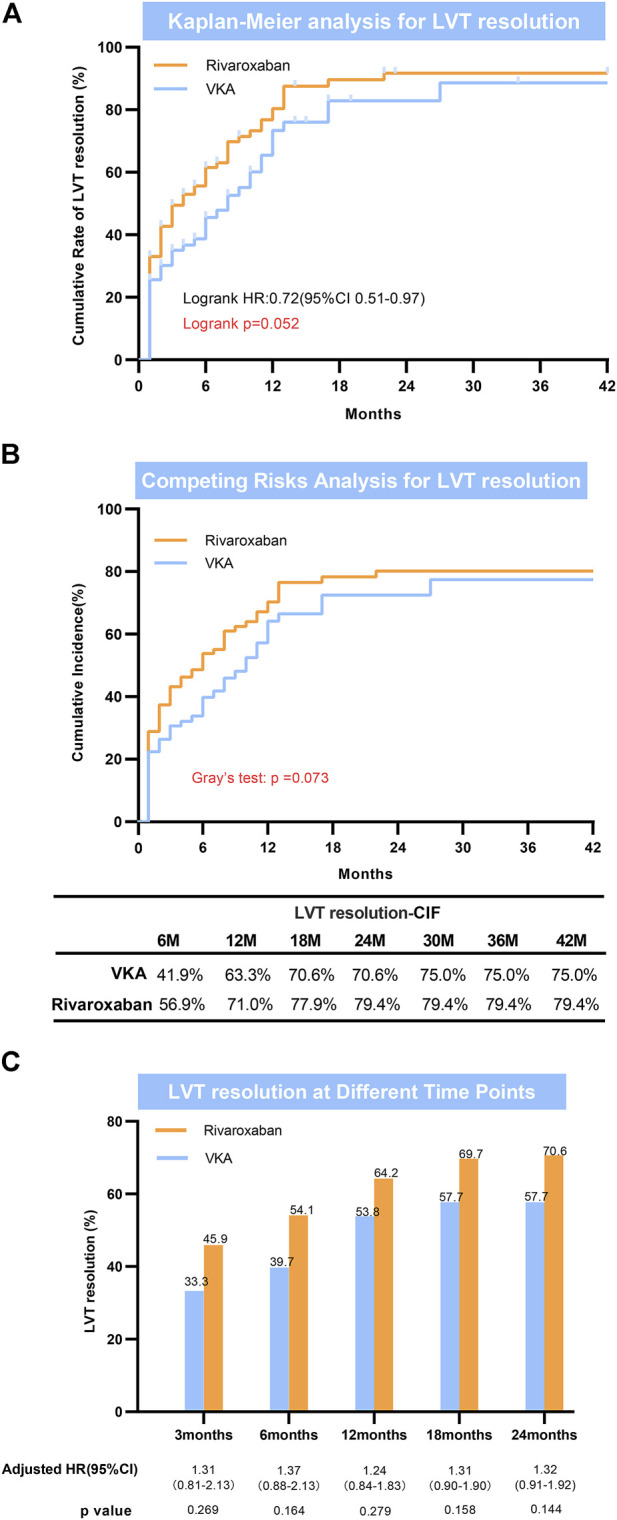
Left ventricular thrombus resolution. **(A)** Kaplan-Meier analysis for LVT resolution; **(B)** Competing risks analysis for LVT resolution; **(C)** LVT resolution at different time points. LVT = left ventricular thrombus.

During the follow-up period (median: 17 months; IQR: 6.0–24.0 months), LV thrombus resolution that confirmed by echocardiography occurred in 46 of 78 (59.6%) patients in the VKAS group and 77 of 109 (70.6%) in the rivaroxaban group (Table 2). Despite the direct observation that the thrombus resolution rates were higher in the rivaroxaban group than in the VKAs group (59.6% vs. 70.6%), the thrombus resolution rates between the two groups did not achieve a statistically significant difference in the adjusted Cox proportional hazards regression model (adjusted HR: 1.303; 95% CI:0.898–1.890; *p* = 0.163) (Table 2). Similar results were obtained in the Fine-Gray model after adjusting for mortality and in the log rank test, with no statistically significant improvement in LV thrombus resolution with rivaroxaban compared to VKAs (Gray’s test *p* = 0.073; log rank *p* = 0.052) (Figure 2A, Figure 2B). In view of these, for a better observation of the difference in the thrombus resolution due to the types of anticoagulants (rivaroxaban vs. VKAs), we compared the thrombus resolution rates according to the time points, and similarly, no statistically significant differences were observed between the two groups (Figure 2C).

**TABLE 2:** Outcomes of Cox Proportional Hazards Regression Analysis in Patients who received Rivaroxaban or VKAs Therapy.

Outcomes	VKAs (*n* = 78)	Rivaroxaban (*n* = 109)	Adjusted HR (95%CI)	*p* value^a^
LV thrombus resolution	46 (59.0)	77 (70.6)	1.303 (0.898–1.890)	0.163
Major adverse cardiovascular events: composite of all-cause mortality, systemic embolism, and rehospitalization	45 (57.7)	54 (49.5)	0.636 (0.418–0.970)	0.035
All-cause mortality	27 (34.6)	31 (28.4)	1.515 (0.891–2.575)	0.125
Systemic embolism	10 (12.8)	5 (4.6)	0.318 (0.108–0.933)	0.037
Rehospitalization for cardiovascular events	21 (26.9)	31 (28.4)	1.064 (0.611–1.852)	0.826
Bleeding events	5 (6.4)	8 (7.3)	1.124 (0.368–3.437)	0.837
Major bleeding	1 (1.3)	0 (0)	—	—
CRNM bleeding	1 (1.3)	0 (0)	—	—
Minor bleeding	3 (3.8)	8 (7.3)	—	—

a P value is for rivaroxaban group as compared with VKAs group.

Abbreviations: LVT, left ventricular thrombus; CRNM, clinically relevant non-major.

### Clinical outcomes

MACEs occurred in 57.7% (*n* = 45) in the VKAs group and 49.5% (*n* = 54) in the rivaroxaban group during the follow-up period (Table 2). A total of 27 patients (34.6%) in the VKAs group and 31 (28.7%) in the rivaroxaban group died. Systemic embolism occurred in 12.8% (*n* = 10) in the VKAs group and 4.6% (*n* = 5) in the rivaroxaban group, respectively. Meanwhile, the incidence of rehospitalization for cardiovascular events was 26.9% (*n* = 21) in the VKAs group and 28.4% (*n* = 31) in the rivaroxaban group. After adjusting for potential confounders in the multivariate Cox proportional hazards regression model, in comparison with the VKAs group, HR for MACEs was lower in the rivaroxaban group (adjusted HR: 0.636; 95% CI:0.418–0.970; *p* = 0.035). For systemic embolism, in the multivariate Cox regression model, relatively low HR was also observed in the rivaroxaban group (adjusted HR:0.318; 95% CI:0.108–0.933; *p* = 0.037), which was consistent with observation in the Fine-Gray model (Gray’s test *p* = 0.041) (Supplementary Table SA4). For all-cause mortality and rehospitalization for cardiovascular events, no statistical differences were observed between the two groups (Table 2, Supplementary Table SA3). Additionally, there was no statistically significant difference in the incidence of ISTH bleeding events between the rivaroxaban and VKAs groups. This result observed in the multivariate COX regression model and the Fine-Gray model was consistent (Table 2, Supplementary Table SA5).

### Exploratory analyses

In the exploratory analysis, subgroups were stratified according to age, sex, types of heart disease, antiplatelet therapy, types of heart failure, and left ventricular ejection fraction. The outcomes were consistent across most subgroups, with no statistically significant interactions, except for in LV thrombus resolution (Supplementary Figures SA1–SA5). In terms of LV thrombus resolution, interaction tests showed a potentially different effects of rivaroxaban in the ischemic heart disease population and the non-ischemic heart disease population (*p* value for interaction = 0.05), but with a wide confidence interval in the non-ischemic heart disease population (HR: 4.215; 95% CI:1.418–12.525) (Supplementary Figure SA1).

## Discussion

Advances in the understanding of the contribution of thrombin generation to thrombosis and the role of DOACs in thrombosis have prompted new anticoagulation paradigms. Since no clinical data on rivaroxaban in HF patients with LV thrombus have been reported, this retrospective study was performed. In this study, we investigated the impact on clinical outcomes of rivaroxaban compared to VKAs in HF patients with LV thrombus in terms of LV thrombus resolution, major adverse cardiovascular events, and bleeding events. In this population, rivaroxaban *versus* VKAs was not found to have a benefit with regard to LV thrombus resolution but observed a decrease in major adverse cardiovascular events and systemic embolism in the setting of the poor prognosis for both groups. Additionally, there was no significant between-group difference in the rate of bleeding events. These findings provide new insights into the anticoagulation management of LV thrombus in the HF setting.

Management of LV thrombus remains a challenge in various clinical settings. In the presence of a definite LV thrombus, no strong trials evidence was available to guide therapeutic strategies or to compare different antithrombotic treatment regimens (Di Odoardo et al., 2021). In this dilemma, the 2017 Canadian Heart Failure Guidelines state that either VKAs or DOACs could be used for LV thrombus on the basis of the lack of trial evidence and mechanism of action (Ezekowitz et al., 2017). Hence, with the available clinical evidence, the management of LV thrombus still presents a considerable uncertainty (Massussi et al., 2021). Some evidence suggested that DOACs, including rivaroxaban, have a quicker resolution rate of LV thrombus than VKAs in the AMI setting, which may be associated with a better clinical benefit (Jones et al., 2021). Furthermore, the No-LVT trial in the all-disease population reported the odds ratio of LV thrombus resolution in the rivaroxaban group was significantly higher than in the warfarin group at 1 month (*N* = 79; odds ratio: 2.813; *p* = 0.03) (Abdelnabi et al., 2021). These studies underscored that rivaroxaban *versus* VKAs perhaps provides a better benefit-risk profile in other clinical settings where LV thrombus exists, yet its role in the clinical setting of HF combined with LV thrombus has not been investigated.

In this study, rivaroxaban *versus* VKAs was not found to have a benefit with regard to LV thrombus resolution; to test the stability of this finding, we used various statistical models, and the finding was generally consistent across statistical models, which was different from some previous LV thrombus-related studies in the non-HF setting (Abdelnabi et al., 2021; Jones et al., 2021; Zhang et al., 2022). The reason for this observation is unclear, which may be that the intrinsic differences of LV thrombus formation in various clinical settings contributed to the complicating interchangeability. Although LV thrombus is considered to be caused primarily by the Virchow triad, left ventricular thrombosis is not equally associated with blood stasis, endocardial alterations, inflammation, and fibrosis in different clinical settings (Massussi et al., 2021). These differences in thrombosis may reasonably translate into differences in antithrombotic activity, resulting in different anticoagulant responsiveness. Notably, although no statistical difference was observed between rivaroxaban and VKAs in terms of LV thrombus resolution, we found a higher rate of resolution with rivaroxaban than with VKAs when observed at all time points. To some extent, it could be considered that rivaroxaban is no worse than VKAs in terms of LV thrombus resolution. However, due to the cohort size and retrospective design, the result should be interpreted with caution.

In the HF population with LV thrombus, prognosis after anticoagulation management is another issue of concern. This study showed that with current guideline-directed anticoagulant therapy, these patients remain at long-term risk for mortality and cardiovascular events. Both groups maintained a high rate of MACEs, but fewer MACE were observed in the rivaroxaban group than in VKAs (49.5% vs. 57.7%, *p* = 0.035), with no significant between-group difference in the rate of bleeding events (7.3% vs. 6.4, *p* = 0.837). This rivaroxaban-related benefit was also reported in other large randomized clinical trials (RCTs) (Mega et al., 2013; Korjian et al., 2018; Branch et al., 2019). A post-hoc analysis of the ATLAS ACS 2-TIMI 51 trial showed that in patients with a history of HF, rivaroxaban at 2.5 mg dose and 5 mg dose significantly reduced the primary efficacy endpoint, cardiovascular mortality, and all-cause mortality without increasing the risk of major bleeding (Korjian et al., 2018). In a post hoc analysis for HF patients in the COMPASS trial, a dose of 2.5 mg rivaroxaban plus aspirin was associated with a greater absolute risk reduction and similar relative risk reduction in MACE compared with non-HF patients (Branch et al., 2019). Furthermore, our study observed that the administration of rivaroxaban in this population reduces systemic embolic events (4.6% vs.12.8%) but has a relatively small impact on death and rehospitalization driven by pump failure-related. This result was similar to the COMMANDER HF results (Zannad et al., 2018; Greenberg et al., 2019), which reported that rivaroxaban in the population in sinus rhythm with heart failure and reduced ejection fraction (HFrEF) prevented thrombo-embolic events, but with little impact on outcome measures that include mortality for the clinical condition of HFrEF; of noted, our study included an all-classified HF population with LV thrombus, whereas the COMMANDER HF investigated population was an HFrEF population in sinus rhythm. In these different HF subsets, the pathophysiological mechanisms leading to patient prognosis are multifaceted, and we need to recognize the impact of the various degrees of left ventricular dysfunction, dilatation, and prothrombotic state associated with HF on the anticoagulant effects of rivaroxaban. However, regardless, clinicians must decide which oral anticoagulant to recommend for these HF patients with LV thrombus. Based on the results of ATLAS ACS 2-TIMI 51, COMPASS, COMMANDER HF, and this study, in the background of current guideline recommendations for administration, perhaps rivaroxaban could be a plausible option, yet more large-scale studies are also needed to confirm the generalizability of our findings, as well as further investigations in pragmatic RCTs in this subset of HF.

### Limitations

First, the retrospective nature of this study predisposes it to selection bias; in particular, LV thrombus was only evaluated by echocardiography. Although we adopted an additional standardized procedure for the final confirmation of LV thrombus to reduce this bias (details in the Supplemental Appendix), this may still contribute to an underestimation of the number of HF patients with LV thrombus. Second, event data was mostly obtained from medical records, which may produce omissions of clinical events. Although we conducted direct telephone follow-up to confirm clinical events, and data was obtained from a highly specialized large-scale heart failure center, which maintained a high level of data recruitment and follow-up, it may still have confounded the analysis of outcomes. Third, we were unable to evaluate the impact of antiplatelet therapy on LV thrombus in detail. Several studies have reported the beneficial effects of antiplatelet therapy on LV thrombus (Altıntaş et al., 2019). Meanwhile, in this study, there was no significant between-group difference in the use of antiplatelet therapy. However, the absence of difference between groups did make our cohort homogenous, allowing us to specifically examine the effect of different anticoagulants. Finally, we are unable to standardize the dose of anticoagulants, but this also reflects the current dilemma of anticoagulation management in this population in the real world. In summary, our findings should not be considered conclusive but rather as a status response to the current anticoagulation management.

## Conclusion

Our data found that in populations with heart failure combined with LV thrombus, the overall prognosis in both the rivaroxaban and VKAs groups was catastrophic. Although rivaroxaban improved the prognosis compared with VKAs to some extent, a considerable need remains for new treatments to improve their clinical course. Further research is needed to provide more robust evidences.

## Data Availability

The original contributions presented in the study are included in the article/Supplementary Material, further inquiries can be directed to the corresponding author.
