# Supplementary material for: Rivaroxaban in heart failure patients with left ventricular thrombus: A retrospective study
Source: Front Pharmacol. 2022 Oct 7;13:1008031. doi: 10.3389/fphar.2022.1008031 (PMC9585209; doi:10.3389/fphar.2022.1008031)
Supplement: Supplementary file 1 [file DataSheet1.DOCX]

**Supplementary Appendix**

**Rivaroxaban in Heart Failure Patients with Left Ventricular Thrombus**

Qian Zhang1, MD, Zhongfan Zhang1, MD, Haikuo Zheng1, MD PhD, Ming Qu2, MD, Shouping Li1, MD, Ping Yang, M.D PhD*, Daoyuan Si1, MD PhD* and Wenqi Zhang1, MD PhD*

^a^Department of Cardiology, China-Japan Union Hospital of Jilin University, Changchun, China

^b^Department of Gastroenterology, Endoscopy Center, China-Japan Union Hospital of Jilin University, Changchun, Jilin, China

**Corresponding authors:**

Wenqi Zhang, MD PhD

Department of Cardiology,China-Japan Union Hospital of Jilin University,Xiantai Street NO.126, Changchun, Jilin, China;

Ph:(+86) :13644407485 Fax: (+86):84995259 Email: [wenqi@jlu.edu.cn](mailto:wenqi@jlu.edu.cn)

Daoyuan Si, MD PhD

Department of Cardiology,China-Japan Union Hospital of Jilin University

Xiantai Street NO.126, Changchun, Jilin, China

Ph:(+86) :13578949866 Fax: (+86):84995233 Email:sidaoyuan@jlu.edu.cn

**Summary**

[1 Definition, Classification and Description of Heart Failure 2](#_Toc7518)

[1.1 Definition of Heart Failure 2](#_Toc20079)

[1.2 Classification of Heart Failure 3](#_Toc9138)

[1.3 Description of chronic heart failure and acute heart failure 3](#_Toc1652)

[2 Left Ventricular Thrombus Echocardiographic Evaluation 4](#_Toc8887)

[2.1 Echocardiographic procedures to confirm LV thrombus 4](#_Toc31687)

[2.2 Non-contrast echocardiography 4](#_Toc13502)

[2.3 Contrast Echocardiography 4](#_Toc5198)

[3 Endpoint Definition and Adjudication 5](#_Toc4464)

[3.1 Endpoint adjudication procedure 5](#_Toc21768)

[3.2 Definition of LV thrombus resolution and persistence 6](#_Toc28152)

[3.3 Definition of major adverse cardiovascular events 6](#_Toc31215)

[3.4 Definition of bleeding events 6](#_Toc3040)

[Reference 7](#_Toc17916)

[eTable 1. Underlying Disease causing Heart Failure 7](#_Toc21386)

[eTable 2. Antiplatelet Therapy in All Heart Failure Patients 8](#_Toc5162)

[eTable 3. Competing Risks Analysis for Rehospitalization for Cardiovascular Events 8](#_Toc32674)

[eTable 4. Competing Risks Analysis for Systemic Embolism 9](#_Toc14757)

[eTable 5. Competing Risks Analysis for Bleeding Events 9](#_Toc18692)

[eTable 6. Details of the clinical covariates included in the multivariate Cox proportional hazard regression model for each outcome 9](#_Toc2716)

[eFigure 1. Subgroup Analysis of LVT resolution 10](#_Toc16104)

[eFigure 2. Subgroup Analysis of Major Adverse Cardiovascular Events 11](#_Toc7738)

[eFigure 3. Subgroup Analysis of All-cause Mortality 12](#_Toc5457)

[eFigure 4. Subgroup Analysis of Systemic Embolism 13](#_Toc5079)

[eFigure 5. Subgroup Analysis of Rehospitalization for Cardiovascular Events 14](#_Toc17426)

**1 Definition, Classification and Description of Heart Failure**

The definition, classification, and description of heart failure(HF) were based on the 2021 ESC Guidelines for the diagnosis and treatment of acute and chronic heart failure(1).

- 1. **Definition of Heart Failure**

According to the 2021 ESC guidelines(1), HF was defined as:

- a clinical syndrome consisting of cardinal symptoms (e.g. breathlessness, ankle swelling, and fatigue) that may be accompanied by signs (e.g. elevated jugular venous pressure, pulmonary crackles, and peripheral oedema) rather than a single pathological diagnosis

It was due to a structural and/or functional abnormality of the heart that results in elevated intracardiac pressures and/or inadequate cardiac output at rest and/or during exercise.

**1.2 Classification of Heart Failure**

According to the 2021 ESC guidelines(1), Heart failure was classified as heart failure with preserved ejection fraction (HFpEF), heart failure with mildly reduced ejection fraction (HFmrEF), and heart failure with reduced ejection fraction (HFrEF). The detailed classification was shown below (**sTable 1**):

| **sTable 1. Definition of HFrEF, HFmrEF, HFpEF** | | | | |
| --- | --- | --- | --- | --- |
| **Type of HF** | | **HFrEF** | **HFmrEF** | **HFpEF** |
| **CRITERIA** | 1 | Symptoms ± Signs^a^ | Symptoms ± Signs^a^ | Symptoms ± Signs^a^ |
|  | 2 | LVEF ≤40% | LVEF 41%-49%^b^ | LVEF ≥50% |
|  | 3 | — | — | Objective evidence of cardiac structural and/or functional abnormalities consistent with the presence of LV diastolic dysfunction/raised LV filling pressures, including raised natriuretic peptides |
| ^a^Signs may not be present in the early stages of HF (especially in HFpEF) and in optimally treated patients.  ^b^For the diagnosis of HFmrEF, the presence of other evidence of structural heart disease (e.g. increased left atrial size, LV hypertrophy or echocardiographic measures of impaired LV filling) makes the diagnosis more likely.  ^c^For the diagnosis of HFpEF, the greater the number of abnormalities present, the higher the likelihood of HFpEF. | | | | |

**1.3 Description of chronic heart failure and acute heart failure**

**Chronic heart failure**(CHF) described those who have had an established diagnosis of HF or who have a more gradual onset of symptoms(at least a 3-month history of heart failure)(1).

**Acute heart failure**(AHF) referred to rapid or gradual onset of symptoms and/or signs of HF, severe enough for the patient to seek urgent medical attention, leading to an unplanned hospital admission or an emergency department visit. AHF may be the first manifestation of HF (new onset) or, more frequently, be due to an acute decompensation of chronic HF(1).

**2 Left Ventricular Thrombus Echocardiographic Evaluation**

**2.1 Echocardiographic procedures to confirm LV thrombus**

Echocardiographic data obtained from medical records or from patients or their family members. The echocardiography results must be independently reviewed by two cardiologists. If there was a difference of view between the cardiologists in the event review, the image was submitted to one other cardiologist for review to ascertain the final outcome.

**2.2 Non-contrast echocardiography**

Non-contrast echocardiography images were acquired in at least 3 (2-, 3-, and 4-chamber) apical views. **LV thrombus** observed by non-contrast echocardiography was defined as (**sFigure 1**):

- a distinct mass of echoes in the left ventricular cavity that was seen clearly throughout the cardiac cycle with a structural texture different from the myocardium;
- this mass to be contiguous with the endocardium in an area of abnormal wall motion;
- could be separated from the underlying endocardium by an endocardial lining.

**2.3 Contrast Echocardiography**

Contrast echocardiography images were acquired in at least 3 (2-, 3-, and 4-chamber) apical views. **LV thrombus** observed by contrast echocardiography was defined as (**sFigure 2**):

- a telltale filling defect sign in a turbid left ventricular cavity;
- this telltale filling defect sign to be contiguous with the endocardium in an area of abnormal wall motion;
- and be avascular and show no contrast enhancement after a high-MI flash impulse, as opposed to tumors.

**3 Endpoint Definition and Adjudication**

**3.1 Endpoint adjudication procedure**

Event data were primarily obtained from medical records, with a small portion obtained through contact with patients and their family members. All event adjudications were performed by a clinical academic group from the Jilin Cardiovascular Disease Research Center according to a pre-specified event definition criteria. The clinical academic group consisted of 3 experts with extensive clinical practice experience in internal medicine and cardiology, who were unknown to the study. Documents considered for endpoint adjudication included: 1) medical history in the medical record; 2) laboratory indicators in the medical record; 3) imaging information in the medical record; and 4) medical documents provided by the patient and his or her family members.

All patient identification information was concealed by the study coordinator from the Jilin Cardiovascular Disease Research Center to ensure an equitable event adjudication. During the adjudication process, two copies of each endpoint package were randomly assigned by the study coordinator to two independent experts for review, and if there was a consensus after review, the adjudication was considered complete, and if there was a disagreement, another independent expert would provide the final adjudication.

**3.2 Definition of LV thrombus resolution and persistence**

LV thrombus status was defined based on the method of Lattuca et al.

**LV thrombus resolution** was defined as the complete disappearance of LVT on all echocardiograms at the last available follow-up visit.

**LV thrombus persistence** was defined as the visibility of thrombus on all echocardiographic views at the last available follow-up visit, which was classified as increased thrombus dimension, stable thrombus, or partial thrombus resolution.

**3.3 Definition of major adverse cardiovascular events**

**Major adverse cardiovascular events** (MACEs) was defined as a composite of all-cause mortality, systemic embolism, rehospitalization for cardiovascular events.

**Systemic embolism** was defined as an arterial embolism resulting in clinical ischemia, including stroke, reinfarction, and acute limb ischemia.

**Rehospitalization for cardiovascular events** was defined as hospitalization caused by worsening or sudden onset of a cardiovascular event.

**Stroke** was defined as a sudden, focal neurologic deficit resulting from a cerebrovascular cause that is not reversible within 24 hours and not due to a readily identifiable cause, such as a tumor or seizure. An event that matched this definition but the symptoms relieved within 24 hours was considered a TIA. Diagnosis required imaging examination (CT, MRI).

**Acute limb ischemia** was defined as a sudden significant worsening of limb perfusion consistent with clinical presentation inculding a new pulse deficit with associated rest pain, pallor, paresthesia, or paralysis.

**3.4 Definition of bleeding events**

Bleeding events were classified as minor bleeding, clinically relevant non-major (CRNM) or major bleeding using the International Society on Thrombosis and Haemostasis (ISTH) definition(2,3).

**Major bleeding** was defined as:

1)fatal bleeding;

2)and/or symptomatic bleeding in a critical area or organ, such as intracranial, intraspinal, intraocular, retroperitoneal, intraarticular or pericardial, or intramuscular with compartment syndrome;

3) and/or bleeding causing a fall in hemoglobin level of 20 g /L (1.24 mmol/L) or more, or leading to transfusion of two or more units of whole blood or red cells.

C**linically relevant nonmajor bleeding** was defined as：

1. requiring medical intervention by a healthcare professional;
2. leading to hospitalization or increased level of care;
3. prompting a face to face (i.e., not just a telephone or electronic communication) evaluation.

**Minor bleeding** was defined as not meeting criteria for major or clinically relevant nonmajor.

**Reference**

1. McDonagh TA, Metra M, Adamo M et al. 2021 ESC Guidelines for the diagnosis and treatment of acute and chronic heart failure. Eur Heart J 2021;42:3599-3726.

2. Schulman S, Kearon C, Subcommittee on Control of Anticoagulation of the S, Standardization Committee of the International Society on T, Haemostasis. Definition of major bleeding in clinical investigations of antihemostatic medicinal products in non-surgical patients. J Thromb Haemost 2005;3:692-4.

3. Kaatz S, Ahmad D, Spyropoulos AC, Schulman S, Subcommittee on Control of A. Definition of clinically relevant non-major bleeding in studies of anticoagulants in atrial fibrillation and venous thromboembolic disease in non-surgical patients: communication from the SSC of the ISTH. J Thromb Haemost 2015;13:2119-26.

**eTable 1. Underlying Disease causing Heart Failure**

| **Underlying Disease causing Heart Failure** | | |
| --- | --- | --- |
| **Underlying disease** | **Number of patients,n** | **Number of patients, %** |
| **Coronary artery disease** | 164 | 82.8% |
| **Dilated cardiomyopathy** | 16 | 8.1% |
| **Hypertrophic cardiomyopathy** | 2 | 1.0% |
| **Myocarditis** | 4 | 2.0% |
| **Valvular heart disease** | 4 | 2.0% |
| **Congenital heart disease** | 2 | 1.0% |
| **Unknown** | 6 | 3.1% |
| **Total** | 198 | 100% |

**eTable 2. Antiplatelet Therapy in All Heart Failure Patients**

| **Antiplatelet Therapy in All Heart Failure Patients** | | | | |
| --- | --- | --- | --- | --- |
|  | **Rivaroxaban** | **VKA** | **Dabigatron** | **None** |
| **No Antiplatelet** | 29 | 15 | 1 | 3 |
| **Aspirin** | 11 | 11 | 1 | 0 |
| **Dual antiplatelet therapy** | 64 | 50 | 0 | 5 |
| **P2Y12 Inhibitor Only** | 5 | 2 | 0 | 1 |
| **Total** | 109 | 78 | 2 | 9 |

**eTable 3. Competing Risks Analysis for Rehospitalization for Cardiovascular Events**

| **Rehospitalization for Cardiovascular Events-CIF** | | | | | | | | |
| --- | --- | --- | --- | --- | --- | --- | --- | --- |
|  | **6M** | **12M** | **18M** | **24M** | **30M** | **36M** | **42M** | **Gray’s test p value** |
| **VKA** | 19.6% | 24.8% | 30.6% | 30.6% | 30.6% | 30.6% | 30.6% | 0.866 |
| **Rivaroxaban** | 20.3% | 26.0% | 29.3% | 34.8% | 34.8% | 34.8% | 34.8% |  |

**eTable 4. Competing Risks Analysis for Systemic Embolism**

| **Systemic Embolism-CIF** | | | | | | | | |
| --- | --- | --- | --- | --- | --- | --- | --- | --- |
|  | **6M** | **12M** | **18M** | **24M** | **30M** | **36M** | **42M** | **Gray’s test p value** |
| **VKA** | 11.9% | 13.6% | 13.6% | 13.6% | 13.6% | 13.6% | 13.6% | 0.041 |
| **Rivaroxaban** | 2.9% | 2.9% | 2.9% | 7.2% | 7.2% | 7.2% | 7.2% |  |

**eTable 5. Competing Risks Analysis for Bleeding Events**

| **Bleeding events-CIF** | | | | | | | | |
| --- | --- | --- | --- | --- | --- | --- | --- | --- |
|  | **6M** | **12M** | **18M** | **24M** | **30M** | **36M** | **42M** | **Gray’s test p value** |
| **VKA** | 3.9% | 3.9% | 3.9% | 6.3% | 6.3% | 6.3% | 6.3% | 0.567 |
| **Rivaroxaban** | 6.5% | 7.8% | 7.8% | 7.8% | 7.8% | 7.8% | 7.8% |  |

**eTable 6. Details of the clinical covariates included in the multivariate Cox proportional hazard regression model for each outcome**

| **clinical covariates included in the multivariate Cox proportional hazard regression model for each outcome** |
| --- |
| **Model 1**  ***Major adverse cardiovascular events:*** Age, LV ejection fraction, NT-proBNP, Types of heart failure(CHF vs. AHF), Diabetes mellitus, Current smoker, Creatinine clearance |
| **Model 2**  ***LV thrombus resolution:*** LV ejection fraction |
| **Model 3**  ***All-cause mortality:*** Age, LV ejection fraction, NT-proBNP, Types of heart failure(CHF vs. AHF) |
| **Model 4**  ***Systemic embolism:***Age, Types of heart failure(CHF vs. AHF) |
| **Model 5**  ***Rehospitalization for cardiovascular events:*** For this outcome, there were no covariates with P≤0.05 in the univariate Cox analysis, so this outcome was finally assessed by a Cox proportional hazard regression model. |
| **Model 6**  ***Bleeding events:*** For this outcome, due to the number of events, so this outcome was finally assessed by a Cox proportional hazard regression model according to the EPV=10 principle. |
| **Noted:** According to clinical weights of covariates and the EPV=10 principle, covariates with P≤0.05 in the univariate models were included in the multivariate Cox proportional hazards regression models to identify the independent effect of anticoagulant types on outcomes. |

**eFigure 1. Subgroup Analysis of LVT resolution**

| **Subgroup Analysis of LVT resolution in Heart Failure Patients**  **Rivaroxaban VS. VKA** |
| --- |
| **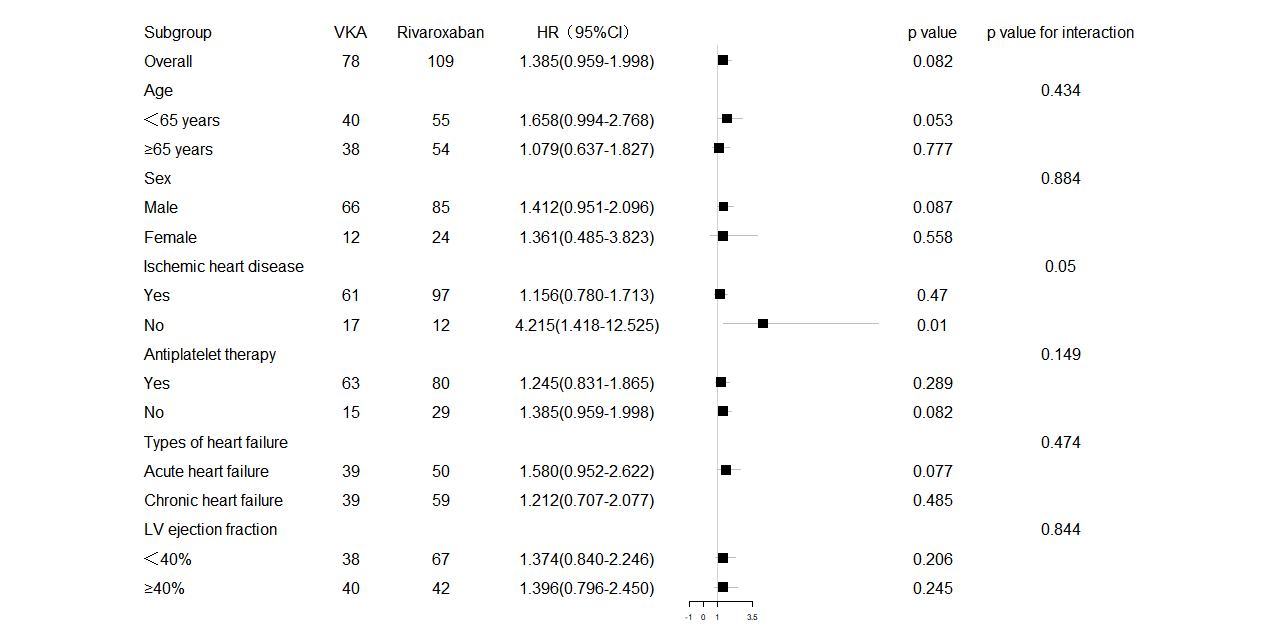** |
| LVT:left ventricular thrombus; LV ejection fraction:left ventricular ejection fraction;  Note: P value for Interaction based on the Cox proportional Hazard joint test. |

**eFigure 2. Subgroup Analysis of Major Adverse Cardiovascular Events**

| **Subgroup Analysis of Major Adverse Cardiovascular Events in Heart Failure Patients**  **Rivaroxaban VS. VKA** |
| --- |
| **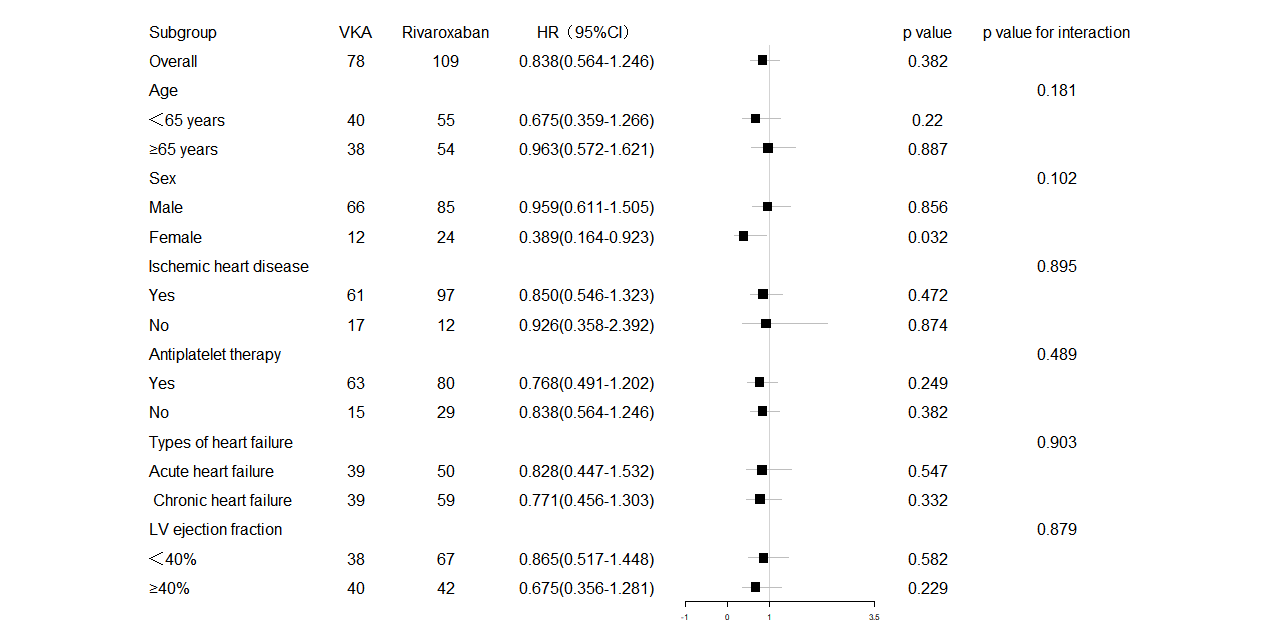** |
| LVT:left ventricular thrombus; LV ejection fraction:left ventricular ejection fraction;  Note: P value for Interaction based on the Cox proportional Hazard joint test. |

**eFigure 3. Subgroup Analysis of All-cause Mortality**

| **Subgroup Analysis of All-cause Mortality in Heart Failure Patients**  **Rivaroxaban VS. VKA** |
| --- |
| **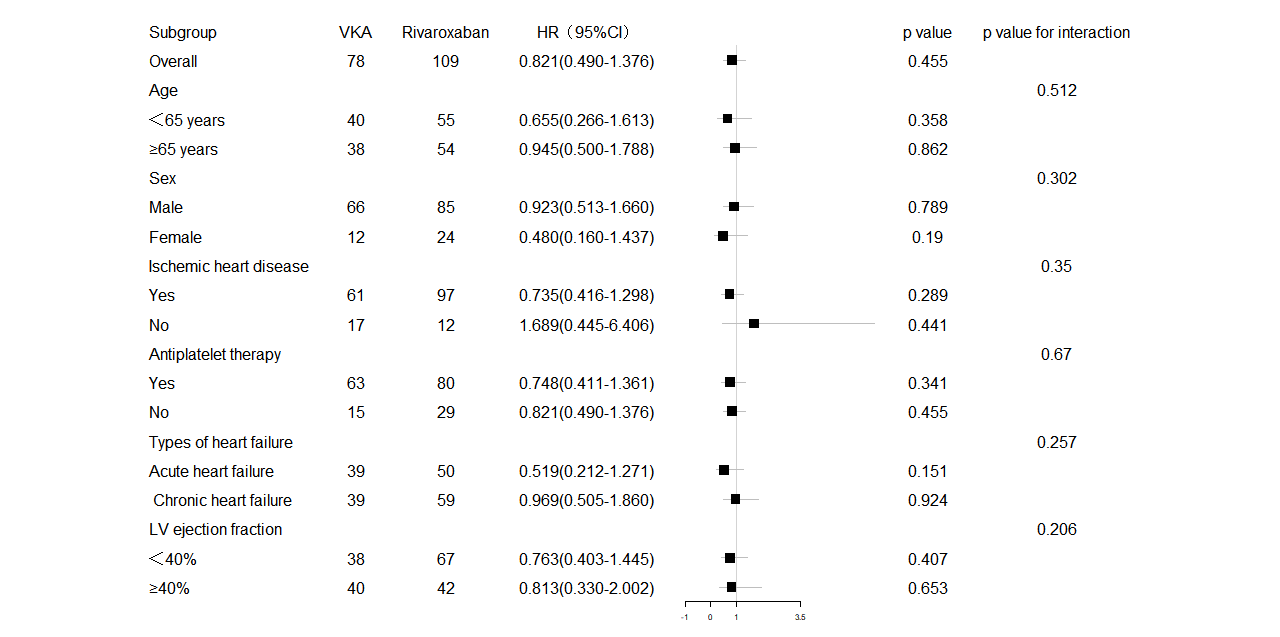** |
| LVT:left ventricular thrombus; LV ejection fraction:left ventricular ejection fraction;  Note: P value for Interaction based on the Cox proportional Hazard joint test. |

**eFigure 4. Subgroup Analysis of Systemic Embolism**

| **Subgroup Analysis of Systemic Embolism in Heart Failure Patients**  **Rivaroxaban VS. VKA** |
| --- |
| **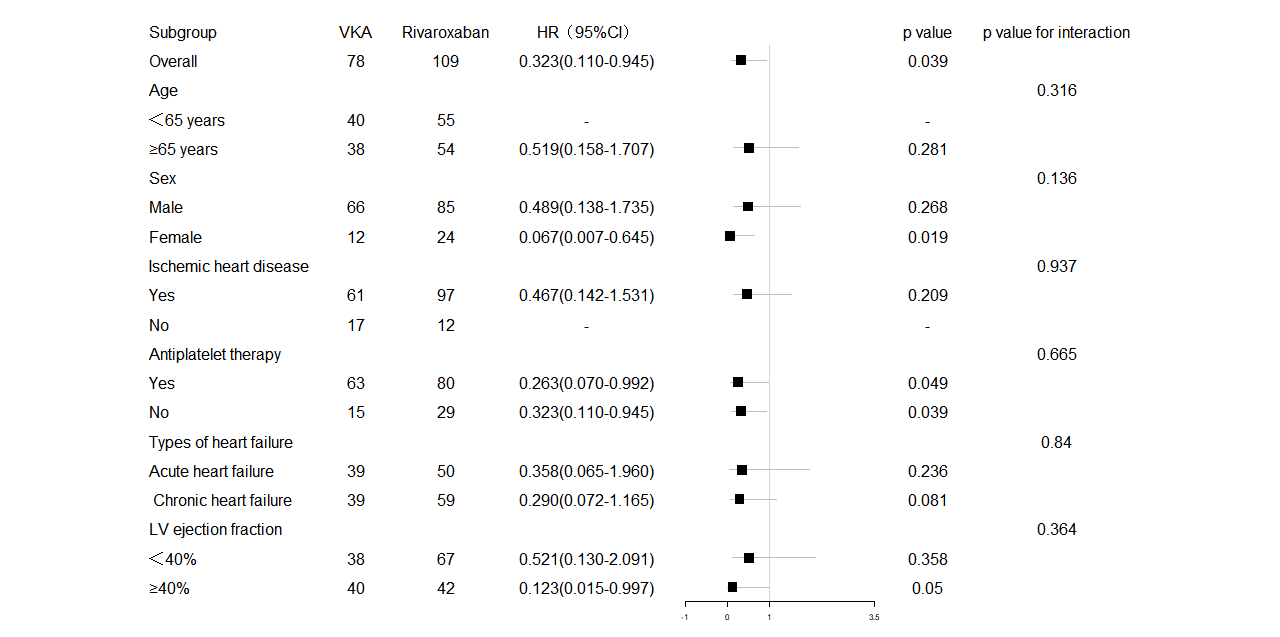** |
| LVT:left ventricular thrombus; LV ejection fraction:left ventricular ejection fraction;  Note: P value for Interaction based on the Cox proportional Hazard joint test. |

**eFigure 5. Subgroup Analysis of Rehospitalization for Cardiovascular Events**

| **Subgroup Analysis of Rehospitalization for Cardiovascular Events**  **Rivaroxaban VS. VKA** |
| --- |
| **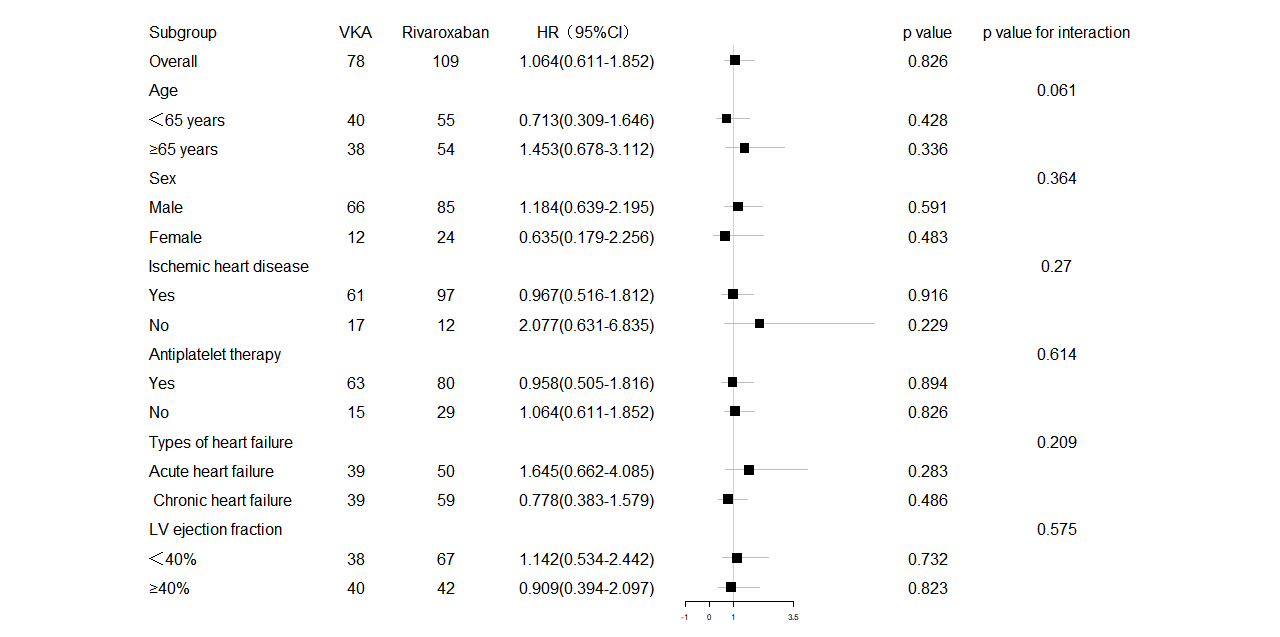** |
| LVT:left ventricular thrombus; LV ejection fraction:left ventricular ejection fraction;  Note: P value for Interaction based on the Cox proportional Hazard joint test. |
